# Supplementary figures and images for: Sandy loam soil maintains better physicochemical parameters and more abundant beneficial microbiomes than clay soil in Stevia rebaudiana cultivation
Source: PeerJ. 2024 Sep 19;12:e18010. doi: 10.7717/peerj.18010 (PMC11416757; doi:10.7717/peerj.18010)

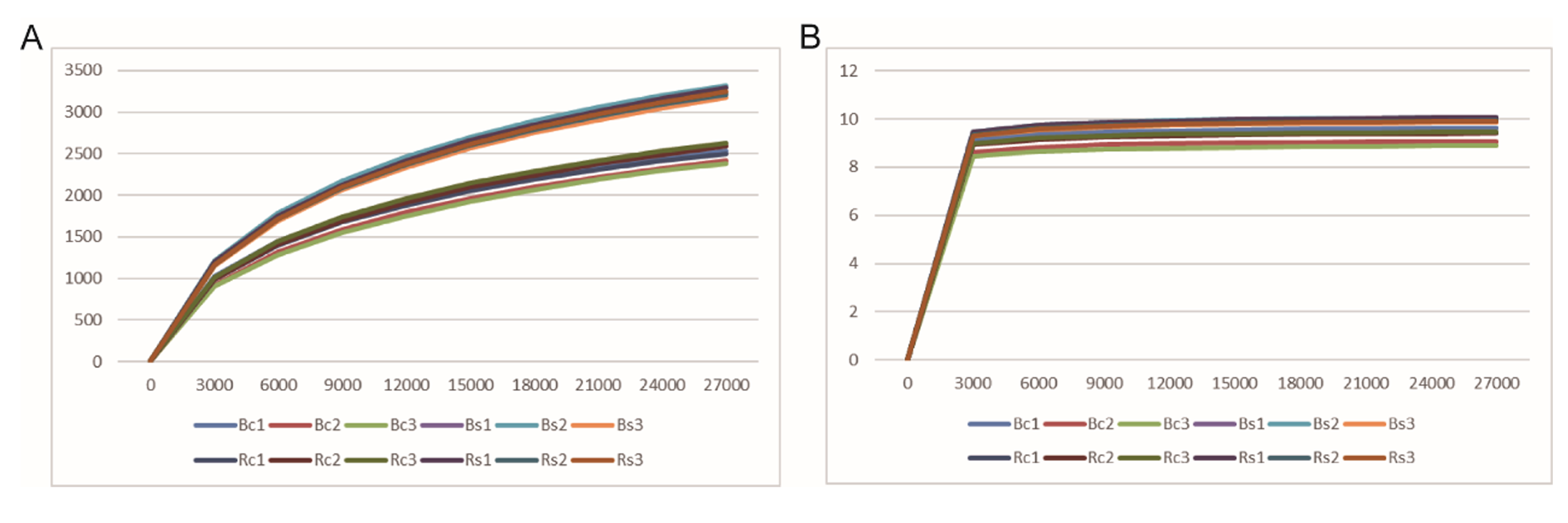

Supplement: Supplemental Information 3 — (A) and Shannon-Wiener indices (B) of bacterial communities based on observed OTUs at a 3% distance. Note: Rs, rhizosphere soil of sandy loam soil; Bs, bulk soil of sandy loam soil; Rc, rhizosphere soil of clay soil; Bc, bulk soil of clay soil. [file peerj-12-18010-s003.jpg]

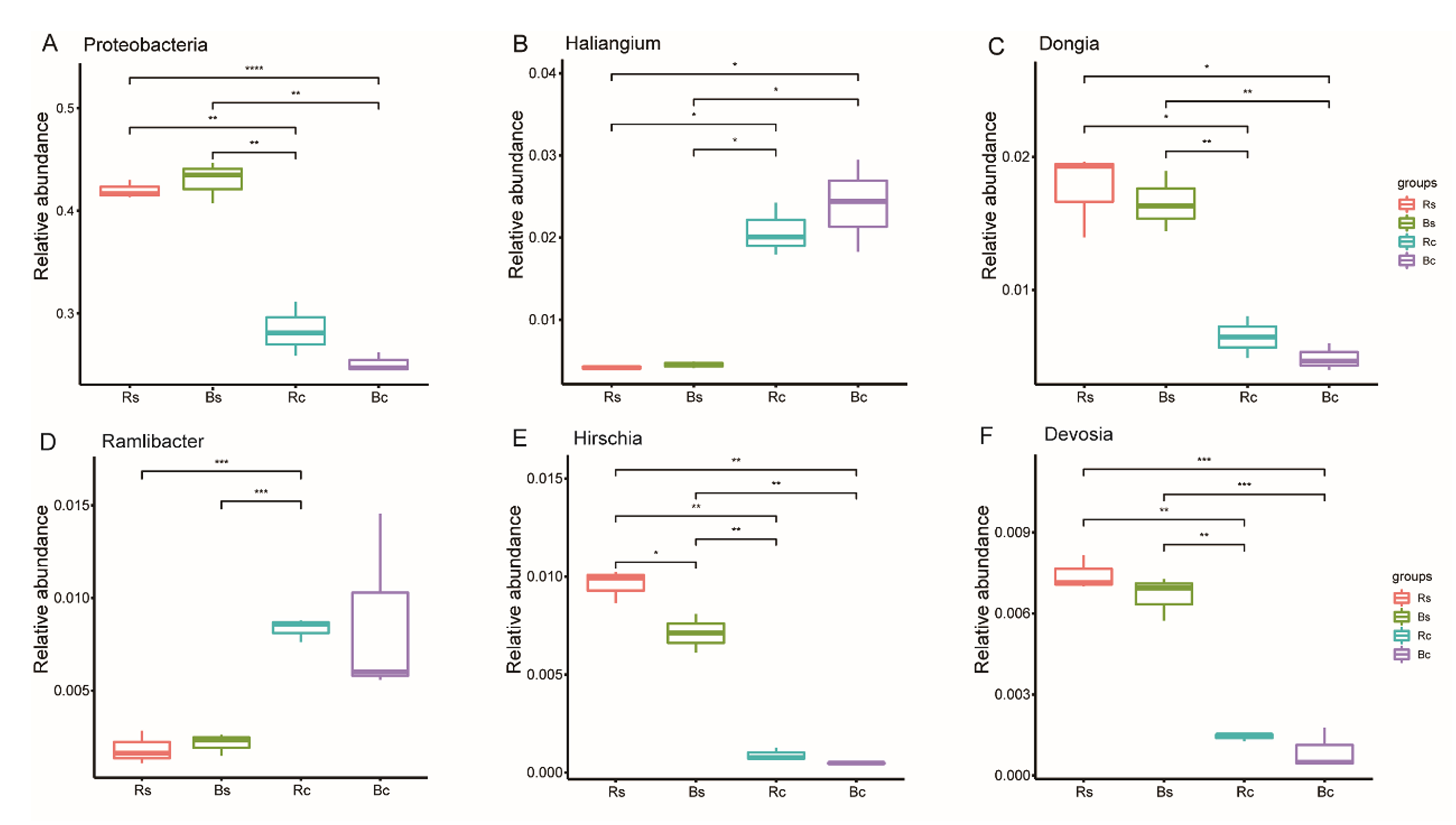

Supplement: Supplemental Information 4 — (A) Proteobacteria. (B) Haliangium. (C) Dongia. (D) Ramlibacter. (E) Hirschia. (F) Devosia. Note: Rs, rhizosphere soil of sandy loam soil; Bs, bulk soil of sandy loam soil; Rc, rhizosphere soil of clay soil; Bc, bulk soil of clay soil. [file peerj-12-18010-s004.jpg]
