# Supplementary material for: Sandy loam soil maintains better physicochemical parameters and more abundant beneficial microbiomes than clay soil in Stevia rebaudiana cultivation
Source: PeerJ. 2024 Sep 19;12:e18010. doi: 10.7717/peerj.18010 (PMC11416757; doi:10.7717/peerj.18010)
Supplement: Supplemental Information 5 — Note: pH, soil pH; OM, organic matter; TN, total nitrogen; AP, available phosphors; AK, available potassium. [file peerj-12-18010-s005.doc]

| Soil texture | Sand particle (%) | Silt particle (%) | Clay particle (%) | Bulk density（g/cm^3^） | pH | OM  （g/kg） | TN  （g/kg） | AP  （mg/kg） | AK  （mg/kg） |
| --- | --- | --- | --- | --- | --- | --- | --- | --- | --- |
| sandy loam soil | 65.43 | 18.22 | 16.35 | 1.32 | 7.93 | 15.79 | 0.93 | 56.02 | 102.67 |
| clay soil | 13.52 | 15.81 | 70.67 | 1.43 | 8.33 | 12.31 | 0.77 | 61.27 | 99.00 |
