# Supplementary material for: Sandy loam soil maintains better physicochemical parameters and more abundant beneficial microbiomes than clay soil in Stevia rebaudiana cultivation
Source: PeerJ. 2024 Sep 19;12:e18010. doi: 10.7717/peerj.18010 (PMC11416757; doi:10.7717/peerj.18010)
Supplement: Supplemental Information 6 — Note: Rs, rhizosphere soil of sandy loam soil; Bs, bulk soil of sandy loam soil; Rc, rhizosphere soil of clay soil; Bc, bulk soil of clay soil. [file peerj-12-18010-s006.doc]

| Sample ID | Raw_tags | Clean_tags | Reads | OTUs |
| --- | --- | --- | --- | --- |
| Rs1 | 102916 | 90207 | 116833 | 3558 |
| Rs2 | 112559 | 99170 | 127283 | 3588 |
| Rs3 | 86625 | 76165 | 99243 | 3399 |
| Bs1 | 102628 | 90366 | 116530 | 3623 |
| Bs2 | 107440 | 93422 | 121657 | 3681 |
| Bs3 | 93264 | 79155 | 106254 | 3327 |
| Rc1 | 94567 | 84671 | 108733 | 2616 |
| Rc2 | 87592 | 78254 | 101382 | 2681 |
| Rc3 | 106464 | 95273 | 122465 | 2863 |
| Bc1 | 91611 | 81744 | 105312 | 2608 |
| Bc2 | 80660 | 72640 | 93566 | 2455 |
| Bc3 | 93635 | 84294 | 108720 | 2618 |
