# Supplementary material for: Sandy loam soil maintains better physicochemical parameters and more abundant beneficial microbiomes than clay soil in Stevia rebaudiana cultivation
Source: PeerJ. 2024 Sep 19;12:e18010. doi: 10.7717/peerj.18010 (PMC11416757; doi:10.7717/peerj.18010)
Supplement: Supplemental Information 7 — Note: pH, soil pH; OM, organic matter; TN, total nitrogen; AP, available phosphors; AK, available potassium; MBC, microbial biomass carbon; MBN, microbial biomass nitrogen; MBP, microbial biomass phosphorus. [file peerj-12-18010-s007.doc]

| Physicochemical properties |  | | | |
| --- | --- | --- | --- | --- |
|  | RDA1 | RDA2 | R^2^ | P |
| pH | 0.9753 | 0.2209 | 0.9191 | **0.002** |
| OM | -0.9964 | 0.0847 | 0.701 | **0.007** |
| TN | -0.9998 | 0.02 | 0.8413 | **0.005** |
| AP | -0.9825 | 0.1863 | 0.9002 | **0.002** |
| AK | 0.7782 | -0.628 | 0.4175 | 0.09 |
| MBC | -0.9859 | 0.1673 | 0.2621 | 0.275 |
| MBN | -0.52 | -0.8541 | 0.0775 | 0.694 |
| MBP | -0.9191 | 0.3941 | 0.7739 | **0.001** |
