# Supplementary material for: Sandy loam soil maintains better physicochemical parameters and more abundant beneficial microbiomes than clay soil in Stevia rebaudiana cultivation
Source: PeerJ. 2024 Sep 19;12:e18010. doi: 10.7717/peerj.18010 (PMC11416757; doi:10.7717/peerj.18010)
Supplement: Supplemental Information 8 — Note: The different lowercase letters in the same column indicate significant differences between different treatments (p < 0.05). [file peerj-12-18010-s008.doc]

| Soil texture | Plant height (cm) | Stem diameter (cm) | Leaf dry weight per plant (g) | Yield (kg/ha^2^) |
| --- | --- | --- | --- | --- |
| Sandy loam soil | 82.60±4.34 a | 1.51±0.04 a | 39.33±1.84 a | 394.60±18.53 a |
| Clay soil | 56.40±3.13 b | 0.95±0.04 b | 26.75±3.48 b | 273.80±13.99 b |
